# Genetic amelioration of fruit and vegetable crops to increase biotic and abiotic stress resistance through CRISPR Genome Editing

**DOI:** 10.3389/fpls.2023.1260102

**Published:** 2023-09-29

**Authors:** Atish Sardar

**Affiliations:** Department of Botany, Jogesh Chandra Chaudhuri College, West Bengal, Kolkata, India

**Keywords:** bacteria, fungi, drought, salinity, fruits, vegetables, CRISPR/Cas9

## Abstract

Environmental changes and increasing population are major concerns for crop production and food security as a whole. To address this, researchers had focussed on the improvement of cereals and pulses and have made considerable progress till the beginning of this decade. However, cereals and pulses together, without vegetables and fruits, are inadequate to meet the dietary and nutritional demands of human life. Production of good quality vegetables and fruits is highly challenging owing to their perishable nature and short shelf life as well as abiotic and biotic stresses encountered during pre- and post-harvest. Genetic engineering approaches to produce good quality, to increase shelf life and stress-resistance, and to change the time of flowering and fruit ripening by introducing foreign genes to produce genetically modified crops were quite successful. However, several biosafety concerns, such as the risk of transgene-outcrossing, limited their production, marketing, and consumption. Modern genome editing techniques, like the CRISPR/Cas9 system, provide a perfect solution in this scenario, as it can produce transgene-free genetically edited plants. Hence, these genetically edited plants can easily satisfy the biosafety norms for crop production and consumption. This review highlights the potential of the CRISPR/Cas9 system for the successful generation of abiotic and biotic stress resistance and thereby improving the quality, yield, and overall productivity of vegetables and fruits.

## Introduction

**Figure 1:**
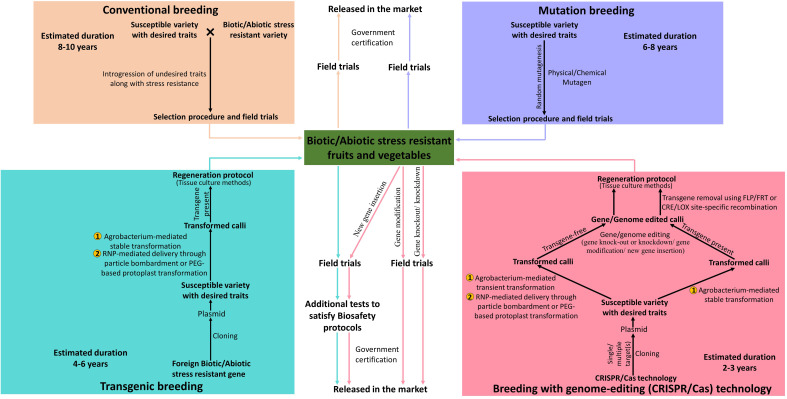
Schematic representation of comparison between traditional, modern and advanced methods of plant breeding for the production of biotic and abiotic stress-resistant vegetable and fruit crops.

Vegetables, fruits, nuts, ornamental, aromatic, and medicinal plants are grouped under horticultural crops. Vegetables and fruits are essential dietary components of our meals as they are excellent sources of carbohydrates, fibres, proteins, vitamins, organic acids, antioxidants, minerals, and trace elements. Climate change and global warming render various dreadful effects on agriculture resulting in loss of crop yield and its nutritional value owing to abiotic factors (heat waves, irregular and uneven precipitation patterns) and biotic factors (increase in plant pathogens and pests). Vegetable and fruit crops are generally more vulnerable to abiotic and biotic stresses leading to huge loss of productivity and nutritional value. Traditional crop breeding such as crossbreeding and mutation-breeding has been successful for a long time to introduce required genetic variations in horticultural crops (Schaart et al., 2016). However, with increasing population, conventional breeding methods, being time-consuming and laborious, are not been able to live up to the growing market demands (Gao, 2021). Transgenic breeding surely provides an alternative by creating genetically modified crops with desired traits in less time but their use is strictly restricted or legally prohibited by government agencies of several countries under safety regulations (Voytas and Gao, 2014). The discovery of genome-editing technology, particularly CRISPR/Cas, has enabled researchers to make precise modifications at specific sites in the DNA to generate horticultural crops possessing novel and desired traits within a short time (Tian et al., 2021) (**Figure 1**).

With the advent of genome editing technology in the 1990s and significant advancements made in the years thereafter, different methods of gene editing have revolutionized the area of research involving functional genomics and crop improvement. Genome editing technology involves the recognition of particular DNA sequences, followed by the induction of double-stranded breaks (DSBs) at specific sites in the targeted DNA using synthetic sequence-specific nucleases (SSNs) (Yin and Qiu, 2019). To maintain genome integrity, cells of almost all living organisms can detect and repair DSBs via either non-homologous end-joining (NHEJ) or homology-directed repair (HDR) (Sonoda et al., 2006). NHEJ is the most common but error-prone DNA-repair process, where DNA ligase IV joins DSBs with minimal DNA end processing in the absence of a repair template, often causing random insertion or deletion of base pairs, leading to a frameshift mutation and thereby resulting in gene knock-out or knockdown. Contrarily, HDR is a high-fidelity repair pathway that uses a homologous repair template DNA to ligate DSBs leading to gene modification or insertion of a new gene (Kumari et al., 2022).

Zinc Finger Nucleases (ZFNs) were the first generation SSNs that were synthesized artificially by the fusion of dimers of Cys2-His2 zinc finger (ZF) domains and *FokI* restriction endonuclease domains and have been successfully used in *Arabidopsis*, *Nicotiana*, and *Zea mays*. However, ZFNs face problems over high-expense, moderate complexity in design, low specificity, difficulty in multiplexing, and consuming time (Kamburova et al., 2017). Later, the identification of transcription activator-like effectors (TALEs) from *Xanthomonas* spp. led to the synthesis of TALENs by joining dimers of TALE domains with *FokI* domains. TALENs have been used with great success in rice, wheat, *Arabidopsis*, potato, and tomato, but high cost, difficulty in multiplexing, complex designing modules, low specificity during screening, and labour-intensiveness pose challenges to their usage (Tavakoli et al., 2021). In 2012, the invention of the CRISPR/Cas9 system from *Streptococcus pyogenes* by Jennifer Doudna and Emmanuelle Charpentier (Jinek et al., 2012), followed by several other discoveries on different CRISPR/Cas systems and rapid advancements and modifications in the technology over the last decade have completely transformed the universe of genome-editing, with multiple gene targeting (multiplexing) becoming a reality. CRISPR/Cas technology possesses several advantages over ZFNs and TALENs for being cheaper, simpler, time-saving, reproducible, and highly efficient in high-yield multiplexing. This article reviews the application of different types of CRISPR/Cas systems in horticultural crops to alleviate biotic and abiotic stresses and discusses challenges and probable solutions.

## CRISPR/Cas genome editing systems

In nature, CRISPR/Cas system serves as an adaptive immune system of bacteria and archaea against the invading foreign DNA originating from plasmids and bacteriophages. The CRISPR/Cas9 system was the first to be discovered and modified into an efficient genome editing tool (Ishino et al., 1987; Jinek et al., 2012). The CRISPR/Cas9 technology comprises two components: the Cas9 endonuclease and the single guide RNA (sgRNA), produced by linking CRISPR RNA (crRNA) with trans-activating crRNA (tracrRNA) and is generally designed with a specific 20-nucleotide spacer sequence complementary to the ‘target’ DNA. For CRISPR/Cas9-mediated genome editing, sgRNA first binds to the DNA target and then recruits Cas9 endonuclease. Next, Cas9 endonuclease thoroughly searches a protospacer-adjacent motif (PAM) sequence, i.e., 5′-NGG-3′ (for *S*. *pyogenes*), in the target DNA and snips the complementary and the non-complementary strands using its two nuclease domains, HNH and RuvC, respectively, producing a blunt DSB (Ran et al., 2013). To expand the range of this technology, several subsequent modifications were introduced into the SpCas9 for recognition of different PAM sequences present in the target DNA, like SpCas9-VQR (5’-NGAN-3′ or 5’-NGNG-3′), SpCas9-EQR (5’-NGAG-3′) SpCas9-VRER (5’-NGCG-3′) and xCas9 (5’-NG-3′, 5’-GAA-3′, and 5’-GAT-3′) (Hu et al., 2018; Yamamoto et al., 2019). Moreover, the discoveries of different PAM site-recognizing Cas9 endonucleases from several other prokaryotes, viz., Cas9 from *Streptococcus thermophilus*, *Neisseria meningitidis*, *Brevibacillus laterosporus*, *Staphylococcus aureus*, and *Geobacillus stearothermophilus* identifies 5’-NNAGAAW-3′ and 5′-NGGNG-3′, 5’-NNNNGATT-3′, 5’-NNNCND-3′, 5’-NNGRRT-3′, and 5′-NNNNCRAA-3′ PAM sites, respectively, have also increased the puissance of the technology (Kim et al., 2021). Another CRISPR/Cas technology, CRISPR/Cas12a (previously known as CRISPR from *Prevotella* and *Francisella*1) system uses a 24-nucleotide spacer complementary sequence for crRNA-DNA/gRNA-DNA binding, doesn’t require a tracrRNA and possesses a smaller endonuclease than most Cas9 orthologs. CRISPR/Cas12a generates staggered-end DSBs after recognizing an AT-rich PAM sequence with a high frequency in the genome and is proving to be a better substitute for CRISPR/Cas9 (Moon et al., 2018). Besides, CRISPR/Cas13a is a single RNA-guided RNA-editing system that needs a crRNA containing a 28-nucleotide spacer complementation sequence to bind adjacent to a protospacer flanking sequence (3′-A/U/C-5′) for Cas13a-mediated target recognition. Cas13a was first identified from *Leptotrichia shahii* and was demonstrated to cleave single-stranded RNAs without the requirement of a tracrRNA (Robertson et al., 2022).

## Biotic stress tolerance in horticultural crops using CRISPR/Cas technology

Biotic stress involves the interaction of plants with plant pathogens such as bacteria, viruses, fungi, oomycetes, nematodes, etc., that hampers the normal growth and development of plants leading to a huge loss in quality and yield (around 30% of crop production worldwide) (van Esse et al., 2020). The generation of disease-resistant crop varieties using CRISPR/Cas technologies has proven to be effective in combating plant diseases in fruits and vegetables (**Table 1**).

**Table 1:** CRISPR/Cas-mediated targeting of genes in fruits and vegetables for imparting resistance against biotic stress.

Pathogen	Disease	Host plant	Technology & Delivery method	Targeted gene	Phenotype	References
**Viruses**						
*Tomato yellow leaf curl virus* (*Begomovirus*)	Tomato yellow leaf curl disease	*Solanum lycopersicum*	CRISPR/Cas9; *Agrobacterium tumefaciens*-mediated transformation	Virus genome (Coat Protein or Replicase)	Resistance to tomato yellow leaf curl disease	Tashkandi et al., 2018
*Tomato yellow leaf curl virus* (*Begomovirus*)	Tomato yellow leaf curl disease	*Solanum lycopersicum*	CRISPR/Cas9; *Agrobacterium tumefaciens*-mediated transformation	Virus genome (Coat Protein and Intergenic Region)	Resistance to tomato yellow leaf curl disease	Faal et al., 2020
*Tomato yellow leaf curl virus* (*Begomovirus*)	Tomato yellow leaf curl disease	*Solanum lycopersicum*	CRISPR/Cas9; *Agrobacterium tumefaciens*-mediated transformation	*Pelo*	Resistance to tomato yellow leaf curl disease	Pramanik et al., 2021
*Tomato mosaic virus* (*Tobamovirus*)	Tomato mosaic disease	*Solanum lycopersicum*	CRISPR/Cas9; *Agrobacterium tumefaciens*-mediated transformation	*DICER-like 2b*	Susceptible to tomato mosaic disease	Wang et al., 2018a
Tobacco mosaic virus (*Tobamovirus*), *Potato virus X* (*Potexvirus*)	Tomato mild mosaic disease	*Solanum lycopersicum*	CRISPR/Cas9; *Agrobacterium tumefaciens*-mediated transformation	*DICER-like 2a* and *2b*	Susceptible to tomato mild mosaic disease	Wang et al., 2018c
*Tomato brown rugose fruit virus* (*Tobamovirus*), *Tomato mosaic virus* (*Tobamovirus*), *Tobacco mosaic virus* (*Tobamovirus*), *Youcai mosaic virus* (*Tobamovirus*)	Multiple viral diseases	*Solanum lycopersicum*	CRISPR/Cas9; *Agrobacterium tumefaciens*-mediated transformation	*Tobamovirus Multiplication 1a*, *1b*, *1c*, *1d*	Resistance to multiple viral diseases	Ishikawa et al., 2022
*Potato virus Y* (*Potyvirus*) PVYN *Cucumber mosaic virus* (*Cucumovirus*)	Tomato mild mosaic disease	*Solanum lycopersicum*	CRISPR/Cas9; *Agrobacterium tumefaciens*-mediated transformation	*Eukaryotic Translation Initiation Factor 4E1*	Resistance to tomato mild mosaic disease	Atarashi et al., 2020
*Pepper Mottle Virus* (*Potyvirus*)	Pepper mottle disease	*Solanum lycopersicum*	CRISPR/Cas9; *Agrobacterium tumefaciens*-mediated transformation	*Eukaryotic Translation Initiation Factor 4E1*	Resistance to pepper mottle disease	Yoon et al., 2020
*Zucchini yellow mosaic virus* (*Potyvirus*), *Papaya ring spot mosaic virus-W* (*Potyvirus*), *Cucumber vein yellowing virus* (*Ipomovirus*)	Multiple viral diseases	*Cucumis sativus*	CRISPR/Cas9; *Agrobacterium tumefaciens*-mediated transformation	*Eukaryotic Translation Initiation Factor 4E*	Resistance to multiple viral diseases	Chandrasekaran et al., 2016
*Potato virus Y* (*Potyvirus*) PVY^O^	Potato mosaic disease	*Solanum tuberosum*	CRISPR/Cas9; RNPs delivery using biolistics or vacuum infiltration methods	*Coilin*	Resistance to potato virus Y strains	Makhotenko et al., 2019
*Potato virus Y* (*Potyvirus*) PVY^O-FL^, PVY^N-Jg^, PVY^N:O-Mb112^	Potato mosaic disease	*Solanum tuberosum*	CRISPR/*Lsh*Cas13; *Agrobacterium tumefaciens*-mediated transformation	Virus genome (Potyviral membrane protein, Cytoplasmic inclusion bodies laminating protein, RNA-dependent RNA polymerase and Coat Protein)	Resistance to potato virus Y strains	Zhan et al., 2019
*Potato virus Y* (*Potyvirus*)	Potato mosaic disease	*Solanum tuberosum*	CRISPR/Cas9; *Agrobacterium tumefaciens*-mediated transformation	*Eukaryotic Translation Initiation Factor 4E*	Resistance to potato virus Y strains	Noureen et al., 2022
*Potato virus Y* (*Potyvirus*)	Potato mosaic disease	*Solanum tuberosum*	CRISPR/Cas9; RNPs delivery via protoplast transformation	*Eukaryotic Translation Initiation Factor 4E1*	Resistance to potato virus Y strains	Lucioli et al., 2022
Synergistic co-infection of *Sweet potato feathery mottle virus* (*Potyvirus*) and *Sweet potato chlorotic stunt virus* (*Crinivirus*)	Sweet potato virus disease	*Ipomoea batatas*	CRISPR/*Rfx*Cas13d; *Agrobacterium tumefaciens*-mediated transient transformation (Heterologous expression in *Nicotiana benthamiana*)	*RNase III endoribonuclease* (pathogenesis-related factor)	Resistance to sweet potato virus disease	Yu et al., 2022
*Cassava brown streak virus* (*Ipomovirus*) *Ugandan cassava brown streak virus* (*Ipomovirus*)	Cassava brown streak disease	*Manihot esculenta*	CRISPR/Cas9; *Agrobacterium tumefaciens*-mediated transformation	*novel Cap-Binding Protein-1* and -*2*	Reduced susceptibility to cassava brown streak disease	Gomez et al., 2019
*South African cassava mosaic virus* (*Begomovirus*)	South African cassava mosaic disease	*Manihot esculenta*	CRISPR/Cas9; RNPs delivery via protoplast transformation	*Ubiquitin E3 Ligase*	Reduced susceptibility to cassava mosaic disease	Chatukuta and Rey, 2020
*Banana streak virus* (*Badnavirus*)	Banana streak disease	*Musa acuminate* × *balbisiana* ‘Gonja Manjaya’ [AAB group]	CRISPR/Cas9; *Agrobacterium tumefaciens*-mediated transformation	Endogenous BSV strain Obinol’Ewai (eBSOLV)	Resistance to banana streak disease	Tripathi et al., 2019
**Bacteria**						
*Pseudomonas syringae* pv. *tomato* DC3000	Bacterial speck	*Solanum lycopersicum*	CRISPR/Cas9; *Agrobacterium tumefaciens*-mediated transformation	*Jasmonate ZIM-domain 2*	Resistance to bacterial speck disease	Ortigosa et al., 2019
*Pseudomonas syringae* pv. *tomato* DC3000 (Δ*avrPto*Δ*avrPtoB*)	Bacterial speck	*Solanum lycopersicum*	CRISPR/Cas9; *Agrobacterium tumefaciens*-mediated transformation	*Acetylenase 1a* and *1b*, *Solyc12g100250, and Solyc12g100270*	Resistance to bacterial speck disease	Jeon et al., 2020
*Pseudomonas syringae* pv. *tomato* DC3000, *Xanthomonas gardneri*, *Xanthomonas perforans*	Multiple bacterial diseases	*Solanum lycopersicum*	CRISPR/Cas9; *Agrobacterium tumefaciens*-mediated transformation	*Downy Mildew Resistance 6-1*	Resistance to diseases caused by *Pseudomonas syringae* pv. *tomato*, *Xanthomonas gardneri*, *X*. *perforans*	Thomazella et al., 2021
*Xanthomonas campestris* pv. *musacearum*	Banana Xanthomonas wilt	*Musa* spp.	CRISPR/Cas9; *Agrobacterium tumefaciens*-mediated transformation	*Downy Mildew Resistance 6*	Resistance to banana Xanthomonas wilt disease	Tripathi et al., 2021
*Xanthomonas citri* subsp. *citri*	Bacterial canker	*Citrus sinensis*	CRISPR/Cas9; *Agrobacterium tumefaciens*-mediated transformation	*Lateral Organ Boundary 1* promoter	Resistance to bacterial canker disease	Peng et al., 2017
*Xanthomonas citri* subsp. *citri*	Bacterial canker	*Citrus sinensis*	CRISPR/Cas9; *Agrobacterium tumefaciens*-mediated transformation	*WRKY22*	Reduced susceptibility to bacterial canker disease	Wang et al., 2019
*Xanthomonas citri* subsp. *citri* ΔpthA4:dCsLOB1.3	Bacterial canker	*Citrus paradisi*	CRISPR/Cas9; *Agrobacterium tumefaciens*-mediated transformation	*Lateral Organ Boundary 1* promoter	Reduced susceptibility to bacterial canker disease	Jia et al., 2016
*Xanthomonas citri* subsp. *citri* ΔpthA4:dCsLOB1.4	Bacterial canker	*Citrus paradisi*	CRISPR/LbCas12a; Agrobacterium *tumefaciens*-mediated transformation	*Lateral Organ Boundary 1* promoter	Reduced susceptibility to bacterial canker disease	Jia et al., 2019
*Xanthomonas citri* subsp. *citri*	Bacterial canker	*Citrus paradisi*	CRISPR/Cas9; *Agrobacterium tumefaciens*-mediated transformation	*Lateral Organ Boundary 1* promoter	Resistance to bacterial canker disease	Jia et al., 2022a
*Xanthomonas citri* subsp. *citri*	Bacterial canker	*Citrus maxima*	CRISPR/ttLbCas12a; *Agrobacterium tumefaciens*-mediated transformation	*Lateral Organ Boundary 1* promoter	Resistance to bacterial canker disease	Jia et al., 2022b
*Erwinia amylovora*	Fire blight	*Malus domestica*	CRISPR/Cas9; RNPs delivery via protoplast transformation	*DspA*/*E-Interacting Proteins from Malus 1, 2 and 4*	Resistance to fire blight disease	Malnoy et al., 2016
*Erwinia amylovora*	Fire blight	*Malus domestica*	CRISPR/Cas9; *Agrobacterium tumefaciens*-mediated transformation	*DspA/E-Interacting Proteins from Malus 4*	Resistance to fire blight disease	Pompili et al., 2020
**Fungi**						
*Oidium neolycopersici*	Powdery mildew	*Solanum lycopersicum*	CRISPR/Cas9; *Agrobacterium tumefaciens*-mediated transformation	*Mildew Resistant Locus O 1*	Resistance to powdery mildew disease	Nekrasov et al., 2017; Pramanik et al., 2021
*Oidium neolycopersici*	Powdery mildew	*Solanum lycopersicum*	CRISPR/Cas9; *Agrobacterium tumefaciens*-mediated transformation	*Powdery Mildew Resistance 4*	Reduced susceptibility to powdery mildew disease	Santillán Martínez et al., 2020
*Pseudoidium neolycopersici*	Powdery mildew	*Solanum lycopersicum*	CRISPR/Cas9; *Agrobacterium tumefaciens*-mediated transformation	*Downy Mildew Resistance 6-1*	Resistance to powdery mildew disease	Thomazella et al., 2021
*Botrytis cinerea*	Grey mould	*Solanum lycopersicum*	CRISPR/Cas9; *Agrobacterium tumefaciens*-mediated transformation	*Mitogen Activated Protein Kinase 3*	Susceptible to grey mould disease	Zhang et al., 2018
*Botrytis cinerea*	Grey mould	*Solanum lycopersicum*	CRISPR/Cas9; *Agrobacterium tumefaciens*-mediated transformation	*MYC2*	Susceptible to grey mould disease	Shu et al., 2020
*Botrytis cinerea*	Grey mould	*Solanum lycopersicum*	CRISPR/Cas9; *Agrobacterium tumefaciens*-mediated transformation	*Acetylenase 1a* and *1b*, *Solyc12g100250, and Solyc12g100270*	Resistance to grey mould disease	Jeon et al., 2020
*Botrytis cinerea*	Grey mould	*Solanum lycopersicum*	CRISPR/Cas9; *Agrobacterium tumefaciens*-mediated transformation	*Pectate Lyase*	Resistance to grey mould disease	Silva et al., 2021
*Botrytis cinerea*	Grey mould	*Solanum lycopersicum*	CRISPR/Cas9; *Agrobacterium tumefaciens*-mediated transformation	*Histone H3 Lysine Methyltransferases Set Domain Group 33* and *34*	Resistance to grey mould disease (double mutants only)	Bvindi et al., 2022
*Botrytis cinerea*	Noble rot	*Vitis vinifera*	CRISPR/Cas9; *Agrobacterium tumefaciens*-mediated transformation	*WRKY52*	Resistance to noble rot disease	Wang et al., 2018b
*Fusarium oxysporum* f. sp. *lycopersici*	Fusarium wilt	*Solanum lycopersicum*	CRISPR/Cas9; *Agrobacterium tumefaciens*-mediated transformation	Unknown function (*Solyc08g075770*)	Resistance to Fusarium wilt disease	Prihatna et al., 2018
*Fusarium oxysporum* f.sp. *niveum*	Fusarium wilt of watermelon	*Citrullus lanatus*	CRISPR/Cas9; *Agrobacterium tumefaciens*-mediated transformation	*Phytosulfokine 1*	Resistance to Fusarium wilt of watermelon disease	Zhang et al., 2020
*Colletotrichum *spp.	Anthracnose	*Capsicum annuum*	CRISPR/Cas9; *Agrobacterium tumefaciens*-mediated transformation	*Ethylene Response Factor* 28	Resistance to anthracnose disease	Mishra et al., 2021
*Erysiphe necator*	Powdery mildew	*Vitis vinifera*	CRISPR/Cas9; RNPs delivery via protoplast transformation	*Mildew Resistant Locus O 7*	Resistance to powdery mildew disease	Malnoy et al., 2016
*Erysiphe necator*	Powdery mildew	*Vitis vinifera*	CRISPR/Cas9; *Agrobacterium tumefaciens*-mediated transformation	*Mildew Resistant Locus O 3*	Resistance to powdery mildew disease	Wan et al., 2020
*Botryosphaeria dothidea*	Ring rot	*Malus domestica*	CRISPR/Cas9; *Agrobacterium tumefaciens*-mediated transformation	*Cyclic Nucleotide-Gated Ion Channels* 2	Resistance to ring rot disease	Zhou et al., 2020
**Oomycetes**						
*Phytophthora capsici*	Blight of tomato	*Solanum lycopersicum*	CRISPR/Cas9; *Agrobacterium tumefaciens*-mediated transformation	*Downy Mildew Resistance 6-1*	Resistance to blight of tomato	Thomazella et al., 2021
*Phytophthora infestans*	Late blight	*Solanum lycopersicum*	CRISPR/Cas9; *Agrobacterium tumefaciens*-mediated transformation	*miR482b and miR482c*	Resistance to late blight disease	Hong et al., 2021
*Phytophthora infestans*	Late blight	*Solanum lycopersicum*	CRISPR/Cas9; *Agrobacterium tumefaciens*-mediated transformation	*MYB transcription factor S2*	Resistance to late blight disease	Liu et al., 2021
*Phytophthora infestans*	Late blight	*Solanum tuberosum*	CRISPR/Cas9; *Agrobacterium tumefaciens*-mediated transformation	*Defense, No Death 1*, *Cryptochrome Interacting Basic helix–loop–helix 1*/ *Homolog of Bee 2 Interacting with IBH 1-like 1*, *Downy Mildew Resistance 6-1*	Resistance to late blight disease	Kieu et al., 2021
*Phytophthora infestans*	Late blight	*Solanum tuberosum*	CRISPR/Cas9; *Agrobacterium tumefaciens*-mediated transformation	*Caffeoyl-CoA O-methyltransferase*	Resistance to late blight disease	Hegde et al., 2021
*Phytophthora infestans*	Late blight	*Solanum tuberosum*	CRISPR/Cas9; RNPs delivery via protoplast transformation	*Signal Responsive 4*	Resistance to late blight disease	Moon et al., 2022
*Phytophthora palmivora*	Blight of papaya	*Carica papaya*	CRISPR/Cas9; *Agrobacterium tumefaciens*-mediated transformation	*Extracellular Cystatin-Like Cysteine Protease Inhibitor 8*	Resistance to blight of papaya disease	Gumtow et al., 2018
*Phytophthora tropicalis*	Black pod	*Theobroma cacao*	CRISPR/Cas9; *Agrobacterium tumefaciens*-mediated transformation	*Non-Expressor of Pathogenesis-Related 3*	Resistance to black pod disease	Fister et al., 2018
*Peronophythora litchii*	Downy blossom blight	*Litchi chinensis*	CRISPR/Cas9	*Pectin Acetylesterase 5*	Less invasive pathogen	Kong et al., 2019
*Peronophythora litchii*	Downy blossom blight	*Litchi chinensis*	CRISPR/Cas9	*Avh142*	Less virulence pathogen	Situ et al., 2020
*Plasmopara viticola*	Downy mildew	*Vitis vinifera*	CRISPR/Cas9; *Agrobacterium tumefaciens*-mediated transformation	*Pathogenesis-Related 4b*	Susceptible to downy mildew disease	Li et al., 2020a

### CRISPR/Cas mediated resistance against viruses

Plant viruses are perilous pathogens causing massive destruction of productivity in horticultural crops. Based on genome composition, plant viruses are grouped into five major categories: double-stranded RNA (dsRNA) viruses, positive sense single-stranded RNA (+ssRNA) viruses, negative sense single-stranded RNA (-ssRNA) viruses, single-stranded DNA (ssDNA) viruses and double-stranded DNA (dsDNA) viruses (Roossinck et al., 2015). Researchers have employed two main strategies to design sgRNAs for engineering virus-resistant plants. The first strategy involves direct targeting of the virus genome. For example, *Tomato yellow leaf curl virus* (TYLCV) is a whitefly-transmitted monopartite *Begomovirus* that causes significant loss of worldwide tomato production. Engineering tomato plants with CRISPR/Cas9 targeting the sequences encoding intergenic region (IR) and coat protein (CP) produced TYLCV-resistant plants where Cas9 was cloned under a virus-inducible *rgsCaM* promoter to overcome any potential off-target effects of Cas9 (Ghorbani Faal et al., 2020). In the plantain banana ‘Gonja Manjaya’, the presence of endogenous *Banana streak virus* (eBSV) as integrative viral elements in the genome limits banana production. CRISPR/Cas9 was utilized to introduce mutations that prevent proper transcription and/or translation into functional viral proteins to produce eBSV-resistant banana plants (Tripathi et al., 2019). Another devastating pathogen, *Potato virus Y* (PVY), belonging to the +ssRNA genome containing genus *Potyvirus*, brings about an 80% decrease in yield and poor tuber quality in potatoes. Targeting the PVY genome with CRISPR/*Lsh*Cas13 system at the sequences encoding potyviral membrane protein (P3), cytoplasmic inclusion bodies laminating protein (CI), RNA-dependent RNA polymerase (NIb) or coat protein (CP) resulted in resistance against multiple strains of PVY in transgenic potato plants (Zhan et al., 2019). To enhance immunity in edited sweet potato plants against sweet potato virus disease, CRISPR/*RfxCas13d* system was used to target the *Sweet potato chlorotic stunt virus* genome encoding pathogenesis-related factor, RNase III endoribonuclease (Yu et al., 2022).

The second strategy requires the manipulation of plant genes responsible for rendering plants susceptible to viruses. For instance, TYLCV-resistant tomatoes were obtained by CRISPR/Cas9-mediated mutagenesis of *SlPelo* that encodes a mRNA surveillance factor (Pramanik et al., 2021). CRISPR/Cas9-mediated multiplexed targeting of susceptibility genes in tomato plants also induces strong resistance to multiple viral diseases. Tomato *Tobamovirus Multiplication 1a-d* are functionally redundant genes essential for *Tobamovirus* multiplication in tomato. The generation of quadruple-mutant of *SlTOM1a-d* through CRISPR/Cas9 multiplexed genome editing exhibited enhanced resistance to *Tobamoviruses* (Ishikawa et al., 2022). RNA viruses hijack host cellular machinery, including *eIF4E*, *eIF4G*, and their isoforms, to complete their life cycle (Sanfaçon, 2015). Different isoforms of *eIF4E* were targeted by CRISPR/Cas9 to generate resistant varieties in tomato, potato, and cucumber against viruses belonging to *Potyviridae* (Chandrasekaran et al., 2016; Atarashi et al., 2020; Yoon et al., 2020; Lucioli et al., 2022; Noureen et al., 2022). Cassava is a tuberous root crop and a staple source of food for sub-Saharan African people. *South African cassava mosaic virus* (SACMV) is a whitefly-transmitted bipartite *Begomovirus* that causes regional pandemics in cassava production in East and Central Africa. Transformation of SACMV-susceptible and -resistant varieties of cassava protoplasts with a ribonucleoprotein complex containing the Cas9 nuclease and sgRNA (CRISPR/Cas9 RNPs) targeting the *Ubiquitin E3 Ligase* gene resulted in less virus titre in protoplasts of susceptible variety, similar to that of the resistant variety protoplasts (Chatukuta and Rey, 2020). Another group used RNP-mediated delivery of CRISPR/Cas9 machinery to enable editing of at least one allele of *Coilin* gene in potato. The edited *Coilin* gene-containing potato plants of the Chicago cultivar showed increased resistance to PVY (Makhotenko et al., 2019).

### CRISPR/Cas mediated resistance against bacteria

Citrus canker, caused by *Xanthomonas citri* subsp. *citri* (*Xcc*), is one of the most economically destructive bacterial disease owing to its appearance at pre- and post-harvest stages. To suppress the immunity of citrus plants, *Xcc* secretes a TALE, PthA4 that binds to effector-binding elements present at the promoter region of *Lateral Organ Boundaries 1* (*LOB1*) (Jia et al., 2016). Citrus canker resistance was first reported in Duncan grapefruit, where CRISPR/Cas9-mediated alteration of PthA4 effector binding elements of one allele of biallelic gene *LOB1* gave partial resistance (Jia et al., 2016), and of two alleles gave complete resistance to *Xcc* (Jia et al., 2022a). In Wanjincheng orange, a multiplexed CRISPR/Cas9 genome editing strategy was applied to delete the entire PthA4 effector binding elements from the promoter region of all the alleles of *CsLOB1* to produce citrus canker-resistant varieties (Peng et al., 2017) In *Arabidopsis*, *Downy Mildew Resistance 6* (*DMR6*) encodes for 2-oxoglutarate Fe(II)-dependent oxygenase that facilitates pathogen infection. In tomato, targeting *DMR6* ortholog, *SlDMR6-1*, via CRISPR/Cas9 resulted in broad-spectrum resistance against multiple pathogens, *Pseudomonas syringae* pv. *tomato* DC3000, *Xanthomonas gardneri*, *X. perforans*, *Pseudoidium neolycopersici*, and *Phytophthora capsici* (Thomazella et al., 2021). Likewise, CRISPR/Cas9-mediated mutagenesis of *MusaDMR6* conferred resistance to *Xanthomonas campestris* pv. *musacearum*, which causes banana Xanthomonas wilt that accounts for huge losses in banana production in East and Central Africa (Tripathi et al., 2021). In apple, two independent research groups using two different transgene-free CRISPR/Cas9 genome editing strategies have demonstrated that editing of one or more genes belonging to the susceptibility gene family, *DspA/E-Interacting Proteins From Malus*, could alleviate fire blight susceptibility to *Erwinia amilovora* (Malnoy et al., 2016; Pompili et al., 2020).

### CRISPR/Cas mediated resistance against fungi

The successful application of CRISPR/Cas technology to mutate the genes responsible for susceptibility has led to the generation of horticultural crops resistant to fungal diseases. *Oidium neolycopersici* and *Erysiphe necator* cause powdery mildew in tomato and grapevine plants, respectively. *Mildew Resistant Locus O* (*Mlo*) encodes a seven-transmembrane domain-containing protein that is conserved throughout angiosperms and negatively regulates immunity to powdery mildew disease (Acevedo-Garcia et al., 2014). In tomato, two independent reports revealed CRISPR/Cas9-mediated targeting of *SlMlo1* confer enhanced resistance to powdery mildew (Nekrasov et al., 2017; Pramanik et al., 2021). Further, (Nekrasov et al., 2017) tested and selfed a specific line of T_0_ transformants to obtain T-DNA-free *slmlo1* plants. Transgene-free powdery mildew-resistant grapevine protoplasts were obtained by knocking out *VvMlo7* via RNP-mediated delivery of CRISPR/Cas9 genome editing machinery (Malnoy et al., 2016). Another report showed CRISPR/Cas9-based alteration of *VvMlo3* was able to produce powdery mildew-resistant grapevine plants (Wan et al., 2020). The multi-host fungal pathogen, *Botrytis cinerea* that causes grey mold disease in tomatoes and noble rot disease in grapevines, poses a serious threat to tomato and grapevine production at both pre- and post-harvest levels. The function of several tomato genes, including the well-characterized genes like *Mitogen Activated Protein Kinase 3* (*MAPK3*), *SlMYC2*, *Pectate Lyase*, *Acetylase 1a* and *1b*, and genes of unknown functions like *Solyc12g100250, and Solyc12g100270*, were identified during *Botrytis cinerea* infection through the generation of deletion mutants by CRISPR/Cas9. While knock-out mutants of *SlMAPK3* and *SlMYC2* were susceptible to *Botrytis cinerea*, loss-of-function of the other above-mentioned genes rendered tomato plants resistant to grey mold disease (Zhang et al., 2018; Jeon et al., 2020; Shu et al., 2020; Silva et al., 2021). In addition, both the single and double knock-out mutants of tomato *Histone H3 Lysine Methyltransferases Set Domain Group33* and *34* (*SDG33* and *SDG34*) produced via CRISPR/Cas9 exhibited alterations in H3K36 and H3K4 methylations. However, only the *sdg33sdg34* plants and not *sdg33* and *sdg34* plants exhibited resistance to *Botrytis cinerea* (Bvindi et al., 2022). The fungal pathogen *Colletotrichum* spp. causes anthracnose of chilli resulting in major pre- and post-harvest losses. In chilli, CRISPR/Cas9-mediated modification *Ethylene Response Factor 28* produced mutant lines with elevated resistance against anthracnose (Mishra et al., 2021). The causal organism of ring rot disease in apple is *Botryosphaeria dothidea*. CRISPR/Cas9-produced apple *Cyclic Nucleotide-Gated Ion Channels 2* knock-out mutant calli exhibited significantly lower growth of *B*. *dothidea*, increased levels of salicylic acid accumulation and elevated expression of several defense-related genes including *Pathogenesis-related* genes, *MdPR1*, *MdPR2*, *MdPR4*, *MdPR5*, *MdPR8*, and *MdPR10a* compared to wild-type calli (Zhou et al., 2020).

### CRISPR/Cas mediated resistance against oomycetes

The most-well studied oomycete, *Phytophthora infestans* is famous for causing the devastating Irish potato famine of the 1840s, killing over a million people. *Phytophthora infestans* causes late blight in potato and tomato plants. Independent studies showing CRISPR/Cas9-targeted mutagenesis of five potato susceptibility genes, *Defense, No Death1*, *CIB1/HBI1-like 1*, *DMR6-1*, *Caffeoyl-CoA O-methyltransferase* and *Signal Responsive 4*, conferred resistance to late blight disease (Hegde et al., 2021; Kieu et al., 2021; Moon et al., 2022). However, knocking-out of *MYB transcription factor S2* by CRISPR/Cas9 produced tomato mutants susceptible to *P. infestans* with increased necrotic cells, lesion sizes, disease index, and reduced expression of defense-related genes, indicating that *SlMYBS2* acts as a positive regulator of resistance to *P. infestans* (Liu et al., 2021). CRISPR/Cas9-engineered cacao plants for the susceptible gene, *Non-Expressor of Pathogenesis-Related 3*, exhibited enhanced resistance to *Phytophthora tropicalis* (Fister et al., 2018).

Editing of the virulent genes in oomycete pathogens provide an alternative strategy to achieve disease resistance. *Phytophthora palmivora* causes worldwide destruction of papaya plantations and reduction in yield. *Extracellular cystatin-like cysteine protease inhibitor 8* is unique to *P. palmivora* and contributes to the virulence of the pathogen by inhibiting papain. CRISPR/Cas9-mediated *PpalEPIC8* editing produced a less virulent version of the pathogen with reduced pathogenicity (Gumtow et al., 2018). Another oomycete, *Peronophythora litchii* causes downy blossom blight of litchi that results in tremendous economic loss in litchi production every year (Situ et al., 2020). Two independent research groups designed CRISPR/Cas9-based modification of two different virulent genes, *Pectin Acetylesterase 5* and *Avh142*, from *P*. *litchii*. *PlPAE5* encodes pectin acetylesterases that cause deacetylation of pectin and *Avh142* is an RXLR effector that induces plant cell-death. Both strategies resulted in the production of less invasive and less virulent variants of *P*. *litchii* that are less capable of infecting litchi plants (Kong et al., 2019; Situ et al., 2020).

## Abiotic stress tolerance in horticultural crops using CRISPR/Cas technology

Plants experience single or multiple abiotic stresses simultaneously, which can lead to 50%-70% loss of crop productivity and poses a direct threat to achieving global food security. (Francini and Sebastiani, 2019). Apart from drought, salinity and temperature, the presence of heavy metals in the soil and excessive use of herbicides and weedicides also contribute to abiotic stress factors (Hamdan et al., 2022). Targeting one or more genes concomitantly utilising CRISPR/Cas technologies could be propitious for engineering abiotic stress-resilient varieties of fruits and vegetables (**Table 2**).

**Table 2:** CRISPR/Cas-mediated targeting of genes in fruits and vegetables for imparting resistance against abiotic stress.

Stress	Plant species	Delivery method	Targeted gene	Phenotype	References
Drought stress	*Solanum lycopersicum*	CRISPR/Cas9; *Agrobacterium tumefaciens*-mediated transformation	*Mitogen Activated Protein Kinase 3*	Sensitive to drought stress	Wang et al., 2017
Drought stress	*Solanum lycopersicum*	CRISPR/Cas9; *Agrobacterium tumefaciens*-mediated transformation	*Non-expressor of Pathogenesis-Related 1*	Sensitive to drought stress	Li et al., 2019
Drought stress	*Solanum lycopersicum*	CRISPR/Cas9; *Agrobacterium tumefaciens*-mediated transformation	*Lateral Organ Boundaries Domain 40*	Tolerance to drought stress	Liu et al., 2020
Drought stress	*Solanum lycopersicum*	CRISPR/Cas9; *Agrobacterium tumefaciens*-mediated transformation	*Gibberellin-Insensitive Dwarf 1a*	Resistance to drought stress	Illouz-Eliaz et al., 2020
Drought stress	*Solanum lycopersicum*	CRISPR/Cas9; *Agrobacterium tumefaciens*-mediated transformation	*Aberrant Growth and Death 2-like Defense Response Protein 1*	Tolerance to drought stress	Wang et al., 2021a
Drought stress	*Solanum lycopersicum*	CRISPR/Cas9; *Agrobacterium tumefaciens*-mediated transformation	*Flavin-dependent Monooxygenase 1*	Sensitive to drought stress	Wang et al., 2021a
Drought stress	*Solanum lycopersicum*	CRISPR/Cas9; *Agrobacterium tumefaciens*-mediated transformation	*Auxin Response Factor 4*	Tolerance to drought stress	Chen et al., 2021
Drought stress	*Solanum lycopersicum*	CRISPR/Cas9; *Agrobacterium tumefaciens*-mediated transformation	*Histone H3 Lysine Methyltransferases Set Domain Group 33* and *34*	Resistance to drought stress (both single and double mutants)	Bvindi et al., 2022
Drought stress	*Solanum tuberosum*	CRISPR/Cas9; *Agrobacterium tumefaciens*-mediated transformation	*FLORE* (long non-coding RNA counterpart of *Cycling DOF Factor 1*)	Sensitive to drought stress	Ramírez Gonzales et al., 2021
Salinity stress	*Solanum lycopersicum*	CRISPR/Cas9; *Agrobacterium tumefaciens*-mediated transformation	*Auxin Response Factor 4*	Tolerance to salinity stress	Bouzroud et al., 2020
Salinity stress	*Solanum lycopersicum*	CRISPR/Cas9; *Agrobacterium tumefaciens*-mediated transformation	*High-affinity K^+^ 20*	Sensitive to salinity stress	Wang et al., 2020
Salinity stress	*Solanum lycopersicum*	CRISPR/ LbCas12a; *Agrobacterium tumefaciens*-mediated transformation	*High-affinity K^+^ Transporter 1;2*	Tolerance to salinity stress	Vu et al., 2020
Salinity stress	*Solanum lycopersicum*	CRISPR/Cas9; *Agrobacterium tumefaciens*-mediated transformation	*Hybrid Proline-rich Protein 1*	Tolerance to salinity stress	Tran et al., 2021
Salinity stress	*Solanum lycopersicum*	CRISPR/Cas9; *Agrobacterium tumefaciens*-mediated transformation	*Salt Overly Sensitive 1*	Sensitive to salinity stress	Wang et al., 2021b
Salinity stress	*Solanum lycopersicum*	CRISPR/Cas9; *Agrobacterium tumefaciens*-mediated transformation	*Mitogen Activated Protein Kinase 3*	Sensitive to salinity stress	Shu et al., 2022
Salinity stress	*Solanum tuberosum*	CRISPR/Cas9; RNPs delivery using biolistics or vacuum infiltration methods	*Coilin*	Tolerance to salinity and osmotic stress	Makhotenko et al., 2019
Salinity stress	*Cucurbita moschata*	CRISPR/Cas9; Agrobacterium rhizogenes-mediated transformation	*Respiratory Burst Oxidase Homolog D*	Sensitive to salinity stress	Huang et al., 2019
Heat stress	*Solanum lycopersicum*	CRISPR/Cas9; *Agrobacterium tumefaciens*-mediated transformation	*Agamous-like 6*	Capable of seedless fruit production under heat stress	Klap et al., 2017
Heat stress	*Solanum lycopersicum*	CRISPR/Cas9; *Agrobacterium tumefaciens*-mediated transformation	*Brassinazole Resistant 1*	Sensitive to heat stress	Yin et al., 2018
Heat stress	*Solanum lycopersicum*	CRISPR/Cas9; *Agrobacterium tumefaciens*-mediated transformation	*Mitogen Activated Protein Kinase 3*	Tolerance to heat stress	Yu et al., 2019
Heat stress	*Solanum lycopersicum*	CRISPR/Cas9; *Agrobacterium tumefaciens*-mediated transformation	*Calcium-Dependent Protein Kinase 28*	Sensitive to heat stress	Hu et al., 2021
Heat stress	*Lactuca sativa*	CRISPR/Cas9; *Agrobacterium tumefaciens*-mediated transformation	*9-cis-Epoxycarotenoid Dioxygenase 4*	Seed germination at high temperature	Bertier et al., 2018
Cold stress	*Solanum lycopersicum*	CRISPR/Cas9; *Agrobacterium tumefaciens*-mediated transformation	*C-Repeat Binding Factors/ Dehydration-Responsive Element Binding Factor 1*	Sensitive to cold stress	Li et al., 2018
Herbicide stress	*Solanum lycopersicum*	CRISPR/Cas9; *Agrobacterium tumefaciens*-mediated transformation	*Acetolactate Synthase 1*	Resistance to herbicide stress	Danilo et al., 2019
Herbicide stress	*Solanum lycopersicum*, *Solanum tuberosum*	CRISPR/Cas9 (cytidine base editors); *Agrobacterium tumefaciens*-mediated transient transformation	*Acetolactate Synthase*	Resistance to herbicide (chlorsulfuron) stress	Veillet et al., 2019
Herbicide stress	*Solanum lycopersicum*	CRISPR/Cas9; *Agrobacterium tumefaciens*-mediated transient transformation	*Acetolactate Synthase*	Resistance to herbicide stress	Yang et al., 2022
Herbicide stress	*Solanum tuberosum*	CRISPR/Cas9; *Agrobacterium tumefaciens*-mediated transformation	*Acetolactate Synthase 1*	Resistance to herbicide stress	Butler et al., 2015
Herbicide stress	*Manihot esculenta*	CRISPR/Cas9; *Agrobacterium tumefaciens*-mediated transformation	*5-Enolpyruvylshikimate-3-phosphate Synthase*	Resistance to herbicide stress	Hummel et al., 2018
Herbicide stress	*Citrullus lanatus*	CRISPR/Cas9; *Agrobacterium tumefaciens*-mediated transformation	*Acetolactate Synthase*	Resistance to herbicide stress	Tian et al., 2021
Herbicide stress	*Malus domestica,* *Pyrus communis*	CRISPR/Cas9; *Agrobacterium tumefaciens*-mediated transformation	*Acetolactate Synthase*	Resistance to herbicide (Chlorsulfuron) stress	Malabarba et al., 2021
Herbicide stress	*Citrus sinensis* × *Poncirus trifoliata*	CRISPR/Cas9; *Agrobacterium tumefaciens*-mediated transformation	*Acetolactate Synthase*	Resistance to herbicide (Imazapyr) stress	Alquézar et al., 2022
High light stress	*Solanum tuberosum*	CRISPR/Cas9; *Agrobacterium tumefaciens*-mediated transformation	*Alternative oxidase*	Resistance to high light stress	Hua et al., 2020

### CRISPR/Cas mediated resistance against drought stress

Plants under drought stress manifest complex symptoms at multiple morphological, physiological, and biochemical levels that consequently lower the quality and yield of the produce. Knocking-out of tomato *Auxin Response Factor 4* (*ARF4*) via CRISPR/Cas9 technology resulted in increased drought resistance and revival ability with induced morphological changes in stomata and vascular bundles, higher content of antioxidant substances, and up-regulated expression of ABA signal transduction pathway genes, like *Scarecrow-Like 3* and *Abscisic acid Insensitive 5* (Chen et al., 2021). Pipecolic acid is known to play an important role in salicylic acid-mediated plant immune response. CRISPR/Cas9-mediated targeting of tomato pipecolic acid biosynthetic pathway gene, *Aberrant Growth and Death2-like Defense Response Protein 1*, has led to increased drought resistance with enhanced CO_2_ assimilation, photosystems activities, antioxidant enzymes activities, ascorbate and glutathione content, and reduced reactive oxygen species accumulation, lipid peroxidation, and protein oxidation than the wild-type tomato plants (Wang et al., 2021a). Interestingly, the *sdg33* and *sdg34* tomato plants were drought-resistant and exhibited high water retention capacity during drought and improved recovery and survival, while *sdg33sdg34* plants were superior drought stress-resistant (Bvindi et al., 2022).

Contrarily, the positive regulators of drought stress were also identified by using CRISPR/Cas technology. For example, CRISPR/Cas9-generated *SlMAPK3* mutant plants were drought-sensitive showing severe wilting symptoms, higher hydrogen peroxide content, and lower antioxidant enzymes activities as compared to the wild-type tomato plants (Wang et al., 2017). Knock-out mutant of another tomato pipecolic acid-related gene, *Flavin-dependent Monooxygenase 1*, exhibited damaged photosystems and impaired antioxidant systems and thus were more sensitive to drought (Wang et al., 2021a). In potato, *Cycling Dof Factor 1* together with a long non-coding RNA (lncRNA) counterpart, *StFLORE*, regulate vegetative reproduction and water homeostasis. CRISPR/Cas9-mediated editing of *StFLORE* promoter region rendered potato mutant plants sensitive to drought due to disruption of stomatal growth and diurnal opening of stomata in an ABA-dependent manner (Ramírez Gonzales et al., 2021).

### CRISPR/Cas mediated resistance against salinity stress

Salinity stress impedes water absorption by altering the osmotic balance between plant roots and surrounding soil and severely affects the yield and quality of crops. CRISPR/Cas9-based precise removal of PRD domain, 8CM domain, or both of *Hybrid Proline-rich Protein 1*, resulted in high salinity tolerance at the germination and vegetative stages of tomato plants (Tran et al., 2021). CRISPR/Cas9 *slarf4* mutants displayed decreased leaf area, CO_2_ assimilation, stomatal conductance, enhanced water use efficiency, and salt and osmotic stress resistance in comparison to wild-type tomato plants (Bouzroud et al., 2020). Transgene-free CRISPR/Cas12a-mediated HDR-based editing of one allele of tomato *High-affinity K^+^ Transporter 1;2* has led to increased salinity stress tolerance, which was completely inherited by theprogeny plants (Vu et al., 2020). Another transgene-free genome editing approach was successful when potato plants manifested tolerance to salt and osmotic stress following CRISPR/Cas9 RNP-based editing of *Coilin* gene (Makhotenko et al., 2019).

Alternatively, CRISPR/Cas9-mediated alteration of *Salt Overlay Sensitive 1* resulted in increased sensitivity to salinity stress in tomato (Wang et al., 2021b). CRISPR/Cas9 *slmapk3* plants, were showcasing salt stress-sensitivity with increased salinity-induced cell death, chlorophyll degradation, and reduced activities of antioxidant enzymes than wild-type tomato plants (Shu et al., 2022). In *Cucurbita moschata*, CRISPR/Cas9-based editing of *Respiratory Burst Oxidase Homolog D* showed salt stress hypersensitive phenotype with a decrease in root apex H_2_O_2_ and K^+^ content (Huang et al., 2019).

### CRISPR/Cas mediated resistance against temperature stress

Temperature stress occurs due to continuous rise or drop in temperature above or below the optimum temperature necessary for plant growth over a period of time. Both heat and cold stresses seriously affect the growth and productivity of crops. Despite being sensitive to drought and salinity stresses, tomato CRISPR/Cas9 *slmapk3* displayed heat stress resistance accompanied by reduced wilting, membrane damage, and enhanced expression of transcripts of heat stress transcription factors and heat shock proteins (Yu et al., 2019). Conversely, CRISPR/Cas9-based modification of *Calcium-Dependent Protein Kinase 28* rendered tomato plants sensitive to heat stress with increased levels of protein oxidation and lower antioxidant enzymes activities than wild-type plants (Hu et al., 2021). In lettuce, CRISPR/Cas9-generated knock-out mutants of *9-cis-Epoxycarotenoid Dioxygenase 4* were capable of seed germination above optimum temperature (Bertier et al., 2018).

Compared to wild-type tomato plants, CRISPR/Cas9-produced *C-repeat Binding Factor 1* knock-out plants demonstrated sensitivity to cold stress accompanied by more severe chilling-injury symptoms, higher electrolyte leakage, and less proline and protein contents, and antioxidant enzymes activities (Li et al., 2018).

### CRISPR/Cas mediated resistance against herbicide stress

Excessive use of herbicides and weedicides causes herbicide stress that leaves a major effect on the physiology of the non-targeted crop plants. CRISPR/Cas technology was applied to prepare herbicide-resistant plants. For example, the tomato *Acetolactate Synthase* (*ALS*) gene was targeted using CRISPR/Cas9 technology to create herbicide-resistant plants (Yang et al., 2022). Moreover, transgene-free HDR-mediated genome editing of *SlALS1* using *Agrobacterium*-assisted CRISPR/Cas9 delivery produced herbicide resistance in tomato plants (Danilo et al., 2019). Transgene-free herbicide-resistant watermelon plants were obtained via CRISPR/Cas9-mediated genome editing of *ClALS* (Tian et al., 2018). Likewise, *PcALS* gene of pear plant was manipulated by CRISPR/Cas9-based base-editing to obtain resistance to the herbicide, chlorosulfuron (Malabarba et al., 2021). Also, CRISPR/Cas9 base-editing of *ALS* gene was used to produce T-DNA-free imazapyr-resistant citrus Carrizo citrange plants (Alquézar et al., 2022).

## Conclusion and future perspective

The application of CRISPR/Cas technology has proven to be quite successful in modern agriculture and in improving the agronomic traits of crops. However, barring tomato plants, the usage of CRISPR/Cas technology is very limited among horticultural crops. Though this technology is now being extended to different horticultural crops, researchers are experiencing several challenges during implementation. The success of CRISPR/Cas application depends on the availability of information about the whole-genome sequence, annotation of genes, and their functions in a particular crop. Therefore, more genome sequencing and functional genomics studies on vegetable and fruit plants are required due to the availability of limited data regarding the identification and characterization of genes controlling important traits, such as quality, yield, biotic, and abiotic stress tolerance (Li et al., 2022). Furthermore, polyploidy is common in vegetable and fruit crops, which make it difficult to study the genome due to their highly diverse and complex nature (Chen et al., 2019). Also, CRISPR/Cas technology has mostly been used to create loss-of-function mutations, which limits its application as gain-of-function mutations in positive regulators controlling important agronomic traits might prove to be advantageous in obtaining desired phenotype(s) in horticultural crops (Kim et al., 2021). Interestingly, a recent report demonstrating successful single or multiplexed activation of gene(s) present in anthocyanin and lignin biosynthesis pathways of pear calli via a third-generation CRISPR activation-mediated gain-of-function mutation system, CRISPR-Act3.0, raised the prospect of further broadening its application to other vegetable and fruit crops (Ming et al., 2022). Another major challenge resides in the perception of common people that do not make any distinction between genetically-modified and genome-edited crops and considers the production and consumption of both crop types to be hazardous to the environment and human health. Additionally, the absence of a common and unified legislative framework that differentiates genome-edited crops from genetically-modified crops and facilitates the production and marketing of genome-edited plants also aggravates the challenge (Bhatta and Malla, 2020). Every nation has drafted its legal framework for the release of genome-edited crops. In general, most countries of North and South America (USA, Canada, Argentina, Colombia), some countries of Asia (India, China, Japan), and Australia consider case-by-case procedures for the release of genome-edited crops and products, while most countries of Europe and New Zealand maintain a conservative approach by adhering to the same restrictive laws framed for production and release of both genetically-modified and genome-edited crops (Wang et al., 2022; Rukavtsova et al., 2023). The presence of the foreign DNA in GMOs is the main reason behind their non-acceptance in society, while the CRISPR/Cas-edited crops could be made transgene-free depending on the mode of delivery of CRISPR/Cas machinery during transformation and the choice of propagation method thereafter. Transgene can easily be removed from CRISPR/Cas-edited crops by self-pollinated sexual reproduction and off-target threat can be reduced by expressing CRISPR/Cas machinery under inducible promoters (Nekrasov et al., 2017; Ghorbani Faal et al., 2020). However, most horticultural crops are propagated vegetatively, especially fruit trees due to their long juvenile period. Moreover, propagation by sexual reproduction would produce transgene-free horticultural crops with undesirable traits as most of the important traits are best expressed when the corresponding genes are present in heterozygous conditions (Tsanova et al., 2021). For the generation of CRISPR/Cas-edited transgene-free horticultural crops, RNP-mediated delivery of CRISPR/Cas machinery through particle bombardment or protoplast transformation using PEG remains the most reliable method, closely followed by *Agrobacterium*-mediated transient expression of CRISPR/Cas cassette. Interestingly, transgene-free genome-edited plants can be produced from *Agrobacterium*-mediated stably transformed horticultural plants by using FLP/FRT and CRE/LOX site-specific recombination systems or by adding two additional Cas9 cleavage target sites at the T-DNA borders (Li et al., 2020b; Wan et al., 2021). The efficiency of transformation and regeneration of horticultural plants in case of both stable and transient transformation are quite low due to their recalcitrance nature towards regeneration protocols. Regeneration process involves tissue culture methods, which are quite difficult, expensive, time-consuming, and labour-intensive (Kumari et al., 2022). Therefore, invention of new technology is required that can bypass the regeneration phase of transformed plants. Plant virus-mediated delivery of CRISPR/Cas machinery has the potency to bypass the regeneration phase as this method takes advantage of virus replication and translocation within plants. The only disadvantage of this process is the limited cargo capacity of most DNA and +ssRNA viruses, which makes delivery of large DNA sequences deletion-prone and thereby preferred replication and translocation of deletion mutant viral vectors over the original vectors (Tsanova et al., 2021). Nevertheless, reports orchestrating the delivery of complete CRISPR/Cas9 machinery by Rhabdovirus and potato virus X and consequent successful editing of *Nicotiana benthamiana* genome enhances the possibility of extending the technology to horticultural plants (Ariga et al., 2020; Ma et al., 2020). Thus, transgene-free CRISPR/Cas-mediated gene/genome-edited mutants of horticultural crops are technically the same as those obtained in nature or from mutation breeding (Li et al., 2022). Conventional breeding and mutation breeding, besides being time-consuming, laborious, and expensive, lack specificity and cause introgression of undesired traits due to linkage drag. Conversely, CRISPR/Cas technology is simple, fast, versatile, and introduces changes in the target gene/genome in a well-defined and efficient manner (Wolter et al., 2019). Moreover, the ability of CRISPR/Cas technology to target multiple genes at one go with maximum precision compared to other existing technologies, and that too in a cost-effective manner, actually makes this technology truly exceptional for genome-edited plant breeding. The upcoming advancements in the CRISPR/Cas system itself and the associated technologies, such as delivery methods with or without regeneration phase, high-throughput sequence-based target analyses, whole genome sequencing, and other omics-based approaches, will simplify the process of identification of key genes controlling biotic and abiotic stress resistance pathways and thereby generation of biotic and abiotic stress-resistant plants.

## Author contributions

AS: Conceptualization, Data curation, Writing – original draft, Writing – review & editing.
