# Corrigendum: Genetic amelioration of fruit and vegetable crops to increase biotic and abiotic stress resistance through CRISPR Genome Editing

**DOI:** 10.3389/fpls.2024.1418620

**Published:** 2024-04-25

**Authors:** Atish Sardar

**Affiliations:** Department of Botany, Jogesh Chandra Chaudhuri College, West Bengal, Kolkata, India

**Keywords:** bacteria, fungi, drought, salinity, fruits, vegetables, CRISPR/Cas9

## Error in Figure/Table

In the published article, there was an error in **Figure 1** as published. The duration of Breeding with genome-editing (CRISPR/Cas) technology is mentioned as 4-6 years. The correct sentence for ‘The duration of Breeding with genome-editing (CRISPR/Cas) technology’ is 2-3 years. The corrected **Figure 1** and its caption appear below.

**Figure 1:**
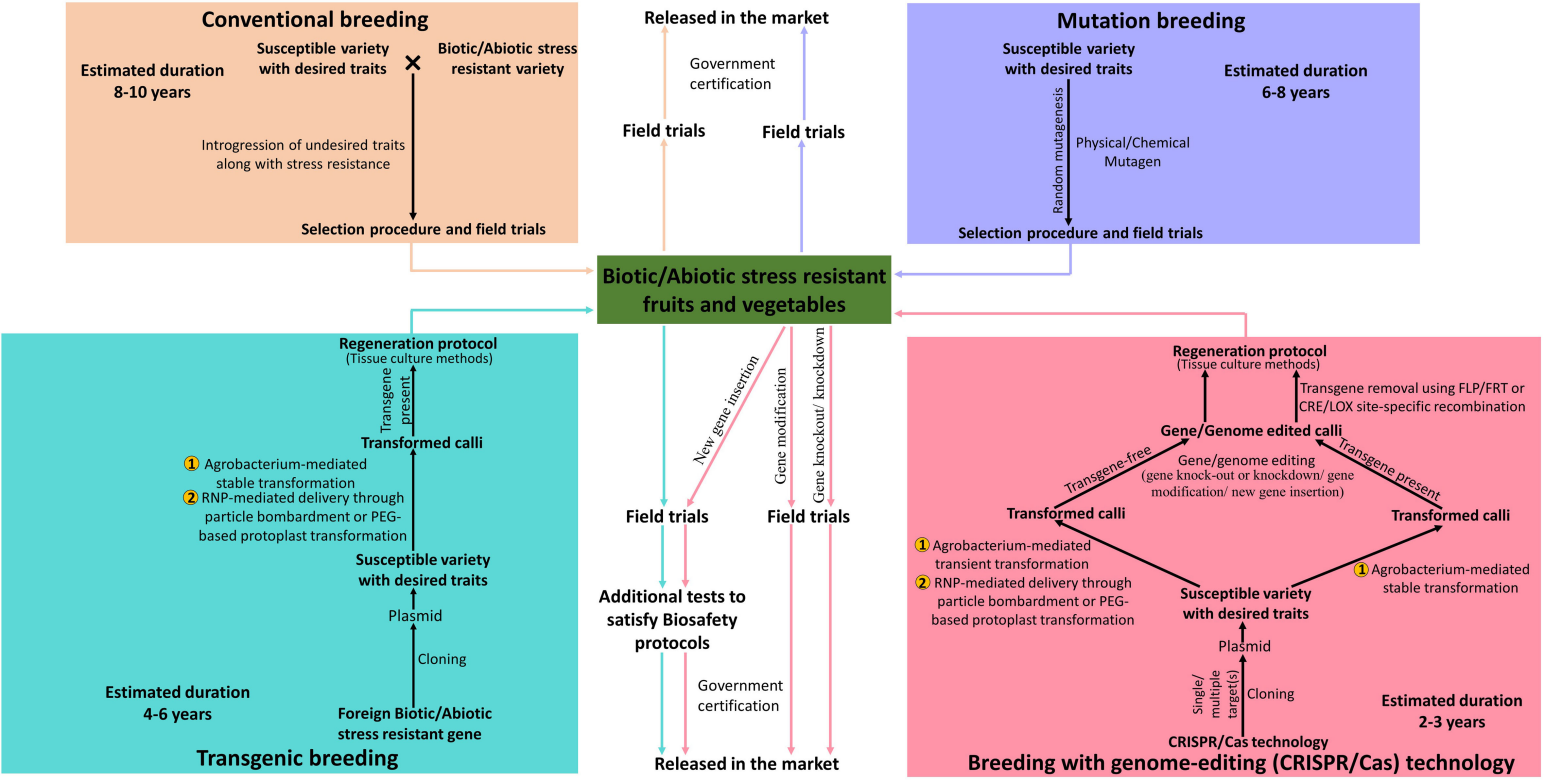
Schematic representation of comparison between traditional, modern and advanced methods of plant breeding for the production of biotic and abiotic stress-resistant vegetable and fruit crops.

The authors apologize for this error and state that this does not change the scientific conclusions of the article in any way. The original article has been updated.

